# Selected ideal natural ligand against TNBC by inhibiting CDC20, using bioinformatics and molecular biology

**DOI:** 10.18632/aging.203642

**Published:** 2021-10-22

**Authors:** Naimeng Liu, Xinhui Wang, Zhu Zhu, Duo Li, Xiaye Lv, Yichang Chen, Haoqun Xie, Zhen Guo, Dong Song

**Affiliations:** 1 Department of Breast Surgery, The First Hospital of Jilin University, Changchun, China; 2 Department of Oncology, First People’s Hospital of Xinxiang, Xinxiang, China; 3 Department of Hematology, The First Clinical Medical School of Lanzhou University, Lanzhou, Gansu, China; 4 Department of General Surgery, China-Japan Friendship Hospital, Beijing, China; 5 Clinical College, Jilin University, Changchun, China

**Keywords:** CDC20, drug development, virtual screening, TNBC, targeted therapy

## Abstract

Object: Find potential therapeutic targets of triple-negative breast cancer (TNBC) patients by bioinformatics. Screen ideal natural ligand that can bind with the potential target and inhibit it by using molecular biology.

Methods: Bioinformatics and molecular biology were combined to analyze potential therapeutic targets. Differential expression analysis identified the differentially expressed genes (DEGs) between TNBC tissues and non-TNBC tissues. The functional enrichment analyses of DEGs shown the important gene ontology (GO) terms and pathways of TNBC. Protein-protein interaction (PPI) network construction screened 20 hub genes, while Kaplan website was used to analyze the relationship between the survival curve and expression of hub genes. Then Discovery Studio 4.5 screened ideal natural inhibitors of the potential therapeutic target by LibDock, ADME, toxicity prediction, CDOCKER and molecular dynamic simulation.

Results: 1,212 and 353 DEGs were respectively found between TNBC tissues and non-TNBC tissues, including 88 up-regulated and 141 down-regulated DEGs in both databases. 20 hub genes were screened, and the higher expression of CDC20 was associated with a poor prognosis. Therefore, we chose CDC20 as the potential therapeutic target. 7,416 natural ligands were conducted to bind firmly with CDC20, and among these ligands, ZINC000004098930 was regarded as the potential ideal ligand, owing to its non-hepatotoxicity, more solubility level and less carcinogenicity than the reference drug, apcin. The ZINC000004098930-CDC20 could exist stably in natural environment.

Conclusion: 20 genes were regarded as hub genes of TNBC and most of them were relevant to the survival curve of breast cancer patients, especially CDC20. ZINC000004098930 was chosen as the ideal natural ligand that can targeted and inhibited CDC20, which may give great contribution to TNBC targeted treatment.

## INTRODUCTION

Triple-negative breast cancer (TNBC), which was defined as no expression of estrogen receptor (ER), progesterone receptor (PR), and human epithelial growth factor receptor 2 (Her2) in breast tumor tissues, accounts for 10%–15% of breast tumor cases [1]. According to the American cancer statistics 2021, breast cancer alone accounts for 30% of female cancers [2]. Although in recent years, early detection, early diagnosis and early treatment improve the cure rate and reduce the mortality rate of the breast cancer, the median survival patients of TNBC was still only 18 months [3]. TNBC as the most aggressive kinds of breast tumor, has a higher recurrence rate and worse prognosis than other types of breast cancer [4]. In recent years, surgery, radiation and chemotherapy are the main treatment of TNBC, however, the patients of TNBC still cannot get effective targeted therapy, because of the tumor heterogeneity [5].

Recently, techniques of bioinformatics were increasingly used to study the signaling pathway of cancers. Some studies have demonstrated that dysregulation of phosphoinositide 3 kinase (PI3K) and AKT signaling pathway can lead to the TNBC [6, 7]. Meanwhile, Tutt et al. also indicated that the mutation of BRCA1 was related to TNBC, and this kind of TNBC patients was especially platinum-sensitive [8]. However, it is still unsatisfactory for patients. Therefore, further study of TNBC molecular mechanisms is still an urgent work.

Also, molecular biology was a hot topic in drug development, and molecular docking and virtual screen were widely used in drug design. By using these methods, we can calculate the pharmacological properties of these ligands [9]. Meanwhile, many natural ligands can be used as lead compounds and converted into new drugs after modification [10]. It can be seen as the first step of drug development. For example, Zhong et al. study suggested that ZINC000003938684 and ZINC000014811844 natural ligands were ideal potential inhibitors of PARP targeting than Olaparib [11]. And Xie et al. found lead compound for the treatment of Alzheimer's disease by virtual screening [12].

**Figure 1:**
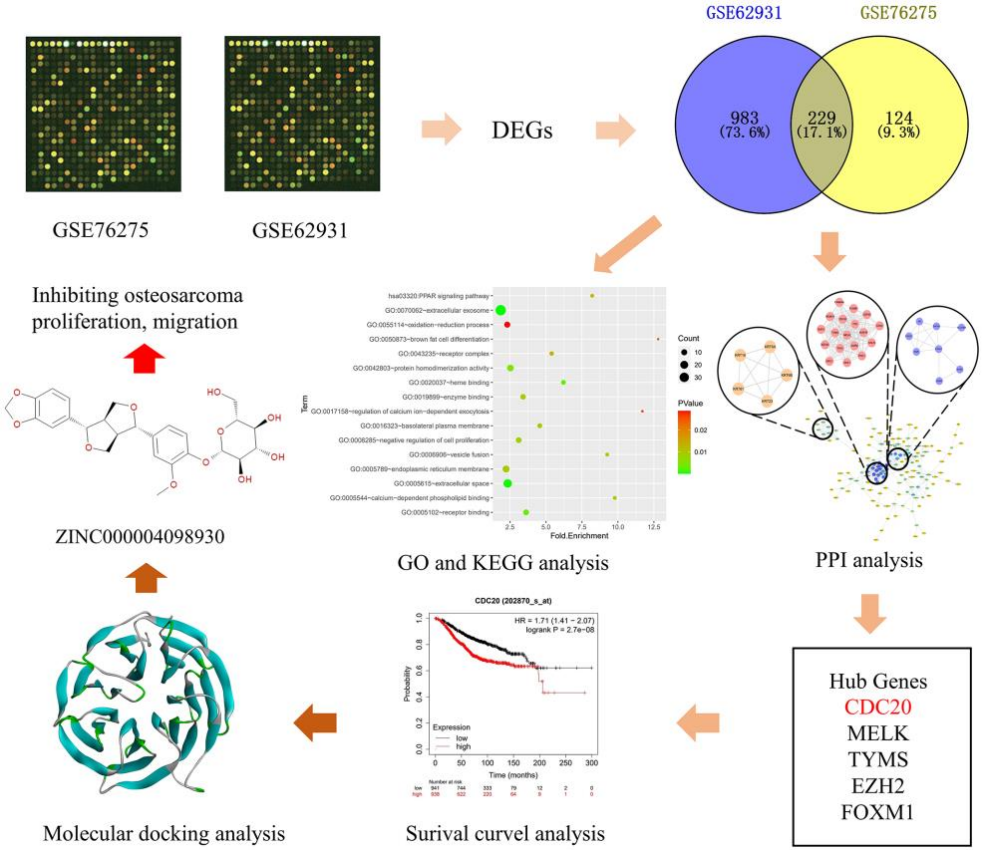
**Frame diagram of this study.** The first image is selected to represent information of tissue datasets from the Gene Expression Omnibus database. Abbreviations: DEG: differentially expressed gene; GO: gene ontology; KEGG: Kyoto Encyclopedia of Genes and Genomes; PPI: protein-protein interaction; CDC20: cell-division cycle protein 20; MELK: maternal embryonic leucine zipper kinase; TYMS: thymidylate synthase; EZH2: enhancer of zeste homolog 2; FOXM1: forkhead box protein M1.

In this study, we combine the bioinformatics with molecular biology to find new way to treat TNBC patients. Firstly, we downloaded GSE62931 and GSE76275 databases and got the differentially expressed genes (DEGs) between TNBC tissues and non-TNBC tissues through these two databases. Then we performed functional and pathway enrichment analysis of these DEGs. Meanwhile, we built protein-protein interaction (PPI) network and analyzed the functions of these DEGs to get 20 most important hub genes. We also evaluated the association of genes expression levels with breast cancer prognosis. Finally, we chose one potential target of TNBC through these 20 hub genes and screened the ideal natural ligands that can combine with it and inhibit it by computational study. In short, this study’s frame diagram was demonstrated in Figure 1, and this study provided a candidate drug to treat TNBC patients.

## METHODS

### Microarray data

We downloaded GSE62931 and GSE76275 databases, which contains 365 samples, from Gene Expression Omnibus (GEO) website (https://www.ncbi.nlm.nih.gov/geo/) [13, 14]. We divided these 2 databases into 2 groups, TNBC tissues and tissues of other type breast cancer. GSE62931 and GSE76275 database were both mRNA expression profiling of TNBC and non-TNBC samples. GSE62931 contains 47 TNBC tissues and 53 other types of breast cancer, while GSE76275 contains 198 TNBC tissues and 67 other types of breast cancer. Meanwhile, we transformed the data of these 2 databases in order to get standardized data, then we compared and analyzed these 2 groups.

### Identification and analysis of DEGs

We used Limma library in R studio and input the code to get the differentially expressed genes (DEGs). The log Fold Change was higher than or equal to 2 and the adjust *P*-value was less than 0.05. Then we used Morpheus website (https://software.broadinstitute.org/morpheus/) to make the Heat maps of these 2 databases. Meanwhile, we used the Limma library again and input the relevant code to get the Volcano plots. These figures were performed with *P*-value <0.05 was defined. The log change >0.5 folds of genes were regarded as up-regulated DEGs and log change <−0.5 folds of genes were regarded as down-regulated DEGs, while others were not-significant DEGs. We also labeled the most significant 20 DEGs in Volcano plots. We also get Venn plots by using https://bioinfogp.cnb.csic.es/tools/venny/index.html to get the DEGs which existed in both GSE62931 and GSE76275 databases.

### Functional and pathway enrichment analyses of DEGs

We used metascape website (https://metascape.org/gp/index.html#/main/step1) to make the Gene Ontology (GO) and Kyoto Encyclopedia of Genes and Genomes (KEGG) pathway enrichment analyses of DEGs. Then we respectively put up-regulated and down-regulated DEGs into Database for Annotation, Visualization and Integrated Discovery (DAVID) website (https://david.ncifcrf.gov/). DAVID website is a database of biological information and an online free analysis software. It provided users extract biological information from a large list of genes or proteins. And The GO analysis included biological processes (BP), molecular functions (MF) and cellular components (CC) of DEGs.

### PPI network construction and selection of module

We used the Search Tool for the Retrieval of Interacting Genes (STRING) website (https://string-db.org/) and Cytoscape software to build protein-protein interaction (PPI) network. Each node in STRING website represents a protein. In STRING website, different isoforms produced by the same gene were combined, and the letter marked on the node is actually the gene symbol of the corresponding gene. The lines between nodes represent interactions between two proteins, and different colors correspond to different types of interactions.

### Prognostic analysis of the most important 20 DEGs

We used Kaplan website (https://kmplot.com/analysis/) to analyze the survival curve. This website is constructed based on gene microarray and RNA-seq data from public databases such as TCGA and GEO. And it assessed the survival impact of more than 50000 genes across 21 types of cancer, including breast cancer. The Kaplan website integrates gene expression information and clinical prognostic value for meta-analysis and the study, discovery and validation of molecular markers related to survival. We input the 20 hub genes and got the overall survival (OS) and recurrence-free survival (RFS) of these 20 hub genes in breast cancer.

### Ligand database and the crystal structure of CDC20

We downloaded the natural products database and the chemical structure of apcin (Protein Data Bank identifier: ZINC000008434966) from ZINC website, which is a free virtual screening database of commercial compounds (http://zinc.docking.org/substances/home/). We screened ideal lead compounds form this database, which contains 17931 ligands.

Meanwhile, we downloaded the crystal structure of CDC20 (4N14) from RCSB PDB website (https://www.rcsb.org/). The RCSB PDB website is powered by the Protein Data Bank archive-information that provided researchers all aspects of biomedicine and agriculture [15].

### ADME and toxicity prediction

We employed the ADME and the TOPKAT to calculate absorption, distribution, metabolism, excretion (ADME) and the toxicity of these compounds, by analyzing chemical structure. These modules were very significant to analyze the safety of ligands, which can save a lot of manpower and material resources [16].

### LibDock and CDOCKER molecular docking

The LibDock module was a simple and fast molecular docking method, which was used to screen large-scale data. While CDOCKER module was a precise docking method, which based on CHARMm flexible docking program. We imported the crystal structure of CDC20 to the Discovery Studio and removed the crystal water and other heteroatoms from it, added, protonation, hydrogen energy minimization and ionization to it. And the apcin’s binding region of CDC20 was chosen as the binding site. Then we ran the LibDock and got 7,416 ligands and their LibDock scores. The top 20 ligands were listed based on the LibDock score [17].

### Molecular dynamic simulation

We selected the ligand-CDC20 complexes’ best binding conformations among these poses by the molecule docking program. We simulated the physiologic environment by adding sodium chloride to the system, which was relaxed by energy minimization in the CHARMm force field. We can use the molecular dynamic simulation to get the potential energy and the RMSD in natural environment.

## RESULTS

### Identification of DEGs

**Figure 2:**
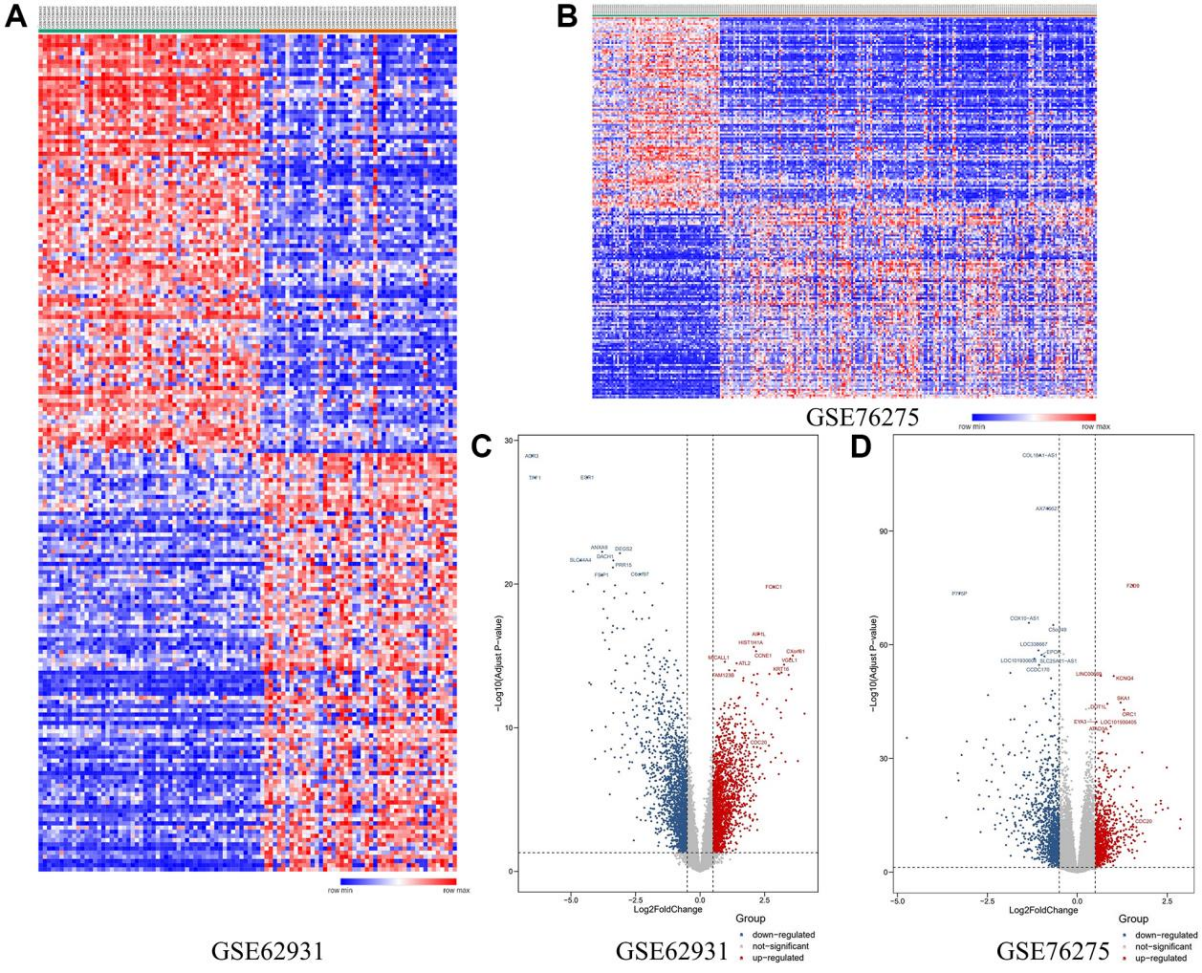
(**A**) The heat-map of DEGs in GSE62931. (**B**) The heat-map of DEGs in GSE 76275. (**C**) Volcano plot of DEGs in GSE62931. (**D**) Volcano plot of DEGs in GSE76275.

Firstly, 2 databases, GSE62931 and GSE76275 were downloaded from GEO website. Then we got 1,212 and 353 DEGs respectively between TNBC tissues and non-TNBC tissues. Heat maps of these two databases’ DEGs expression were shown in Figure 2A and 2B. Besides, the Volcano plots showed the relationship between the Fold change and *P*-value of each DEGs (Figure 2C and 2D). These DEGs were divided into 3 kinds, down-regulated, up-regulated and not-significant DEGs.

**Figure 3:**
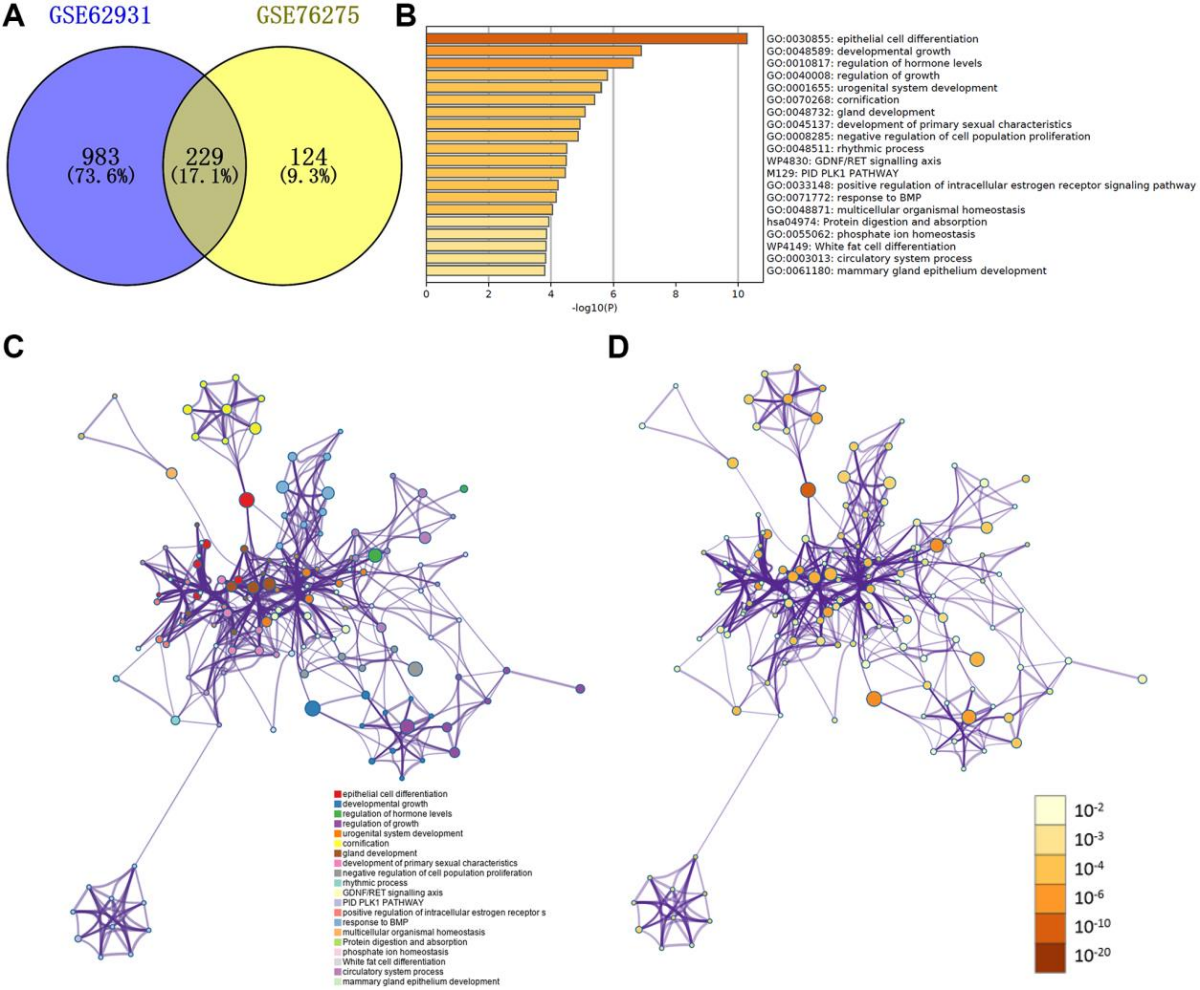
(**A**) Venn plot of DEGs in GSE62931 and GSE76275. (**B**) GO terms and enriched KEGG pathways of the DEGs. (**C**) DEGs colored by cluster ID. DEGs in the same cluster ID nodes are closely related to each other. (**D**) DEGs colored by *P*-value. Terms with more significant *P*-values contain more genes.

Then the Venn plot showed that 229 DEGs in these two databases, and there are 88 up-regulated and 141 down-regulated DEGs (Figure 3A and Supplementary Figure 1A and 1B).

### Functional and pathway enrichment analyses

We used metascape website to make the functional and pathway enrichment analysis (Figure 3B–3D, Supplementary Table 1). The enrichments of DEGs were mostly in ‘epithelial cell differentiation’, ‘developmental growth’, ‘regulation of hormone levels’, ‘urogenital system development’ and ‘regulation of growth’. Figure 3C and 3D were respectively colored by cluster ID and *P*-value.

Meanwhile, we respectively put the up-regulated and down-regulated DEGs into DAVID website, and made the functional enrichment analysis again (Supplementary Figure 1C and 1D). The GO analysis results of the biological processes (BP), molecular functions (MF) and cellular components (CC) illustrated that up-regulated DEGs in TNBC patients were mainly enriched in ‘cytoplasm’, ‘identical protein binding’ and ‘mitotic nuclear division’. And the down-regulated genes of TNBC patients in BP, MF and CC were mainly enriched in ‘negative regulation of cell proliferation’, ‘heme binding’ and ‘extracellular exosome’.

In addition, the KEGG pathway enrichment analysis demonstrated up-regulated genes were enriched in ‘cell cycle’, ‘glioma’, ‘biosynthesis of amino acids’ and ‘biosynthesis of antibiotic’. Meanwhile, the down-regulated genes of TNBC patients were enriched in the ‘PPAR signaling pathway’ by the KEGG pathway enrichment analysis.

### PPI network construction and the selection of module

**Figure 4:**
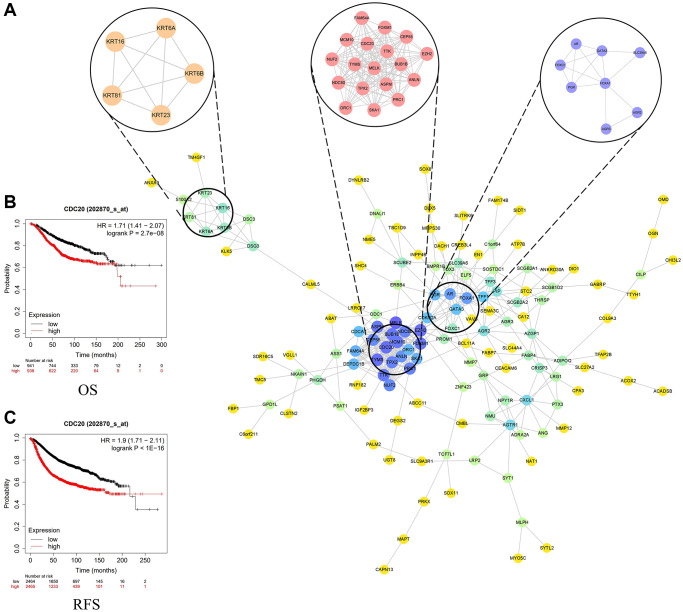
(**A**) Top three modules from the protein-protein interaction network. (**B**) Overall survival in patients with breast cancer based on the expression of CDC20. (**C**) Recurrence-free survival in patients with breast cancer based on the expression of CDC20.

We input 229 DEGs into STRING and Cytoscape software and built PPI network (Figure 4A). Based on the degrees, we get the top 20 genes as the hub genes, including MELK, CDC20, EZH2, TYMS, MCM10, BUB1B, FOXM1, ASPM, TTK, TPX2, NDC80, PRC1, CEP55, NUF2, ANLN, FOXA1, AR, SKA1, FAM64A and DEPDC1B (Table 1). Then we selected the most important 3 modules (Supplementary Table 2). The module 1 contains 18 genes, including CDC20. The module 2 contains 5 genes and the module 3 contains 8 genes.

**Table 1:** Detailed information of the hub genes.

**Name**	**Degree**	**MNC**
MELK	22	21
CDC20	21	20
EZH2	21	19
TYMS	21	18
MCM10	20	20
BUB1B	20	20
FOXM1	20	19
ASPM	20	19
TTK	20	19
TPX2	19	19
NDC80	19	18
PRC1	19	17
CEP55	18	18
NUF2	18	18
ANLN	17	17
FOXA1	16	14
AR	16	10
SKA1	14	14
FAM64A	14	14
DEPDC1B	13	12

We also made the functional enrichment analysis of these 3 modules and got the Supplementary Figure 2. The genes of module 1 were mainly enriched in ‘sister chromatid cohesion’, ‘cell division’ and ‘mitotic nuclear division’, while genes of module 2 were mostly enriched in ‘intermediate filament’, ‘keratin filament’ and ‘structural molecule activity’. And as for module 3, genes were mostly enriched in ‘transcription factor binding’, ‘transcriptional activator activity’ and ‘transcription regulatory region DNA binding’.

### Survival analysis of hub genes

We put these 20 hub genes into Kaplan website and performed the survival curve analysis. The results illustrated that the overall survival (OS) of TNBC patients with higher expression 14 hub genes were shorter than those with lower expression 14 hub genes (*P* < 0.01), especially CDC20 (Figure 4B). Likewise, the recurrence-free survival (RFS) of TNBC patients with lower expression 18 hub genes were longer than TNBC patients with higher expression 18 hub genes (*P* < 0.01), especially CDC20 (Figure 4C). On the contrary, the RFS and OS of TNBC patients with higher expression AR genes were longer than those with lower expression AR genes (Supplementary Figures 3–6). In short, most of hub genes were associated with poor prognosis of TNBC patients. Cell-division cycle protein 20 homologue (CDC20) as one of the most significant hub gene and an important gene in module 1, was shown to be a great therapeutic target of TNBC patients [18]. Therefore, we chose CDC20 as targeted site for further study.

### ADME and toxicity properties of CDC20 inhibited ligands

**Figure 5:**
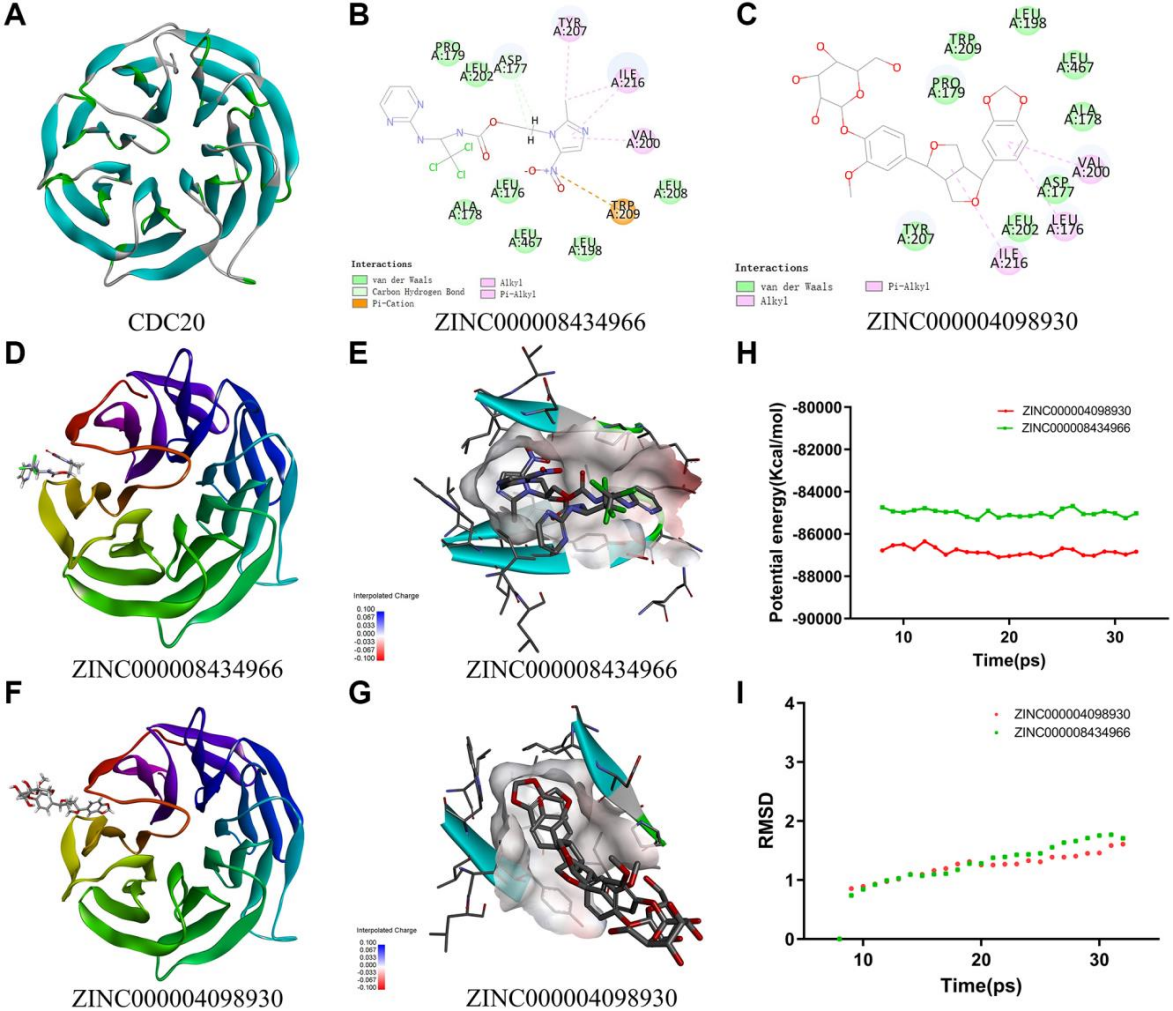
(**A**) The crystal structure of CDC20. (**B**) Schematic of intermolecular interaction of ZINC000008434966 with CDC20. (**C**) Schematic of intermolecular interaction of ZINC000004098930 with CDC20. (**D**) The crystal structure of CDC20 with ZINC000008434966. (**E**) The charge between the ZINC000008434966 and CDC20 surface. (**F**) The crystal structure of CDC20 with ZINC000004098930. (**G**) The charge between the ZINC000004098930 and CDC20 surface. (**H**) Potential energy of the compounds ZINC000008434966 and ZINC000004098930, Average backbone root-mean-square deviation. (**I**) RMSD of the compounds ZINC000008434966 and ZINC000004098930, root-mean-square deviation.

We downloaded a natural database from ZINC database, which contains 17,931 ligands, to screen potential CDC20 targeted inhibitor. Meanwhile, we chose apcin, a CDC20 targeted inhibitor, as the reference drug [19]. We also downloaded the crystal structure of CDC20 protein and chemical structure of reference drug, apcin (Figure 5A and Supplementary Figure 7A). 7,416 ligands were indicated to bind firmly with CDC20 protein through the LibDock screening. We listed the top 20 ligands in Supplementary Table 3 based on the LibDock score. Then we analyzed the Pharmacologic properties of these top 20 ligands by ADME and Toxicity Prediction (Tables 2 and 3). Among these ligands, owing to the non-hepatotoxicity, more solubility level and less carcinogenicity than apcin, ZINC000004098930 (Supplementary Figure 7B) was selected as the lead compounds for further study.

**Table 2:** Adsorption, distribution, metabolism, and excretion properties of compounds.

**Number**	**Compounds**	**Solubility Level**	**BBB Level**	**CYP2D6**	**Hepatotoxicity**	**Absorption Level**	**PPB Level**
1	ZINC000001577210	2	1	1	1	0	0
2	ZINC000014455080	3	1	1	1	0	0
3	ZINC000085826837	2	4	1	1	2	1
4	ZINC000013130935	1	4	1	0	2	0
5	ZINC000004098930	3	4	1	0	2	1
6	ZINC000028968101	1	4	0	0	3	0
7	ZINC000028968107	1	4	0	0	3	0
8	ZINC000044281738	0	4	1	0	3	0
9	ZINC000038143594	3	4	1	1	3	1
10	ZINC000044086691	1	4	1	1	3	0
11	ZINC000002526388	2	4	0	0	0	0
12	ZINC000049784088	4	4	1	1	3	1
13	ZINC000004098458	3	4	1	1	3	1
14	ZINC000004098459	3	4	1	1	3	1
15	ZINC000014951634	3	4	1	1	3	1
16	ZINC000011616636	2	4	1	1	3	1
17	ZINC000008214697	2	4	1	1	3	0
18	ZINC000031165470	4	4	1	1	3	1
19	ZINC000017654900	2	4	1	0	2	1
20	ZINC000014951658	3	4	1	1	3	1
21	ZINC000008434966	2	4	1	0	1	1

Abbreviations: BBB: blood-brain barrier; CYP2D6: cytochrome P-450 2D6; PPB: plasma protein binding. Aqueous-solubility level: 0, extremely low; 1, very low, but possible; 2, low; 3, good. BBB level: 0, very high penetrant; 1, high; 2, medium; 3, low; 4, undefined. CYP2D6 level: 0, noninhibitor; 1, inhibitor. Hepatotoxicity: 0, nontoxic; 1, toxic. Human-intestinal absorption level: 0, good; 1, moderate; 2, poor; 3, very poor. PPB: 0, absorbent weak; 1, absorbent strong.

**Table 3:** Toxicities of compounds.

**Number**	**Compounds**	**Mouse NTP**		**Rat NTP**		**Ames**	**DTP**
		**Female**	**Male**	**Female**	**Male**		
1	ZINC000001577210	0	0.173	0	0.952	0	0.04
2	ZINC000014455080	0.889	0	0.001	0.681	0.018	1
3	ZINC000085826837	0.186	1	1	0.998	0	1
4	ZINC000013130935	0	1	1	0.011	0	0
5	ZINC000004098930	0.302	0	1	0	0	1
6	ZINC000028968101	1	0.021	0.06	0.997	1	1
7	ZINC000028968107	1	0.021	0.06	0.997	1	1
8	ZINC000044281738	0	0	1	0	0.007	0
9	ZINC000038143594	0.061	0	0.274	0.088	0	1
10	ZINC000044086691	1	0	0.987	0.998	0.004	1
11	ZINC000002526388	0.999	0.041	0	0.999	0.999	0.745
12	ZINC000049784088	0.995	0	0	0.008	1	1
13	ZINC000004098458	0.005	0	0.988	0.003	0	1
14	ZINC000004098459	0.005	0	0.988	0.003	0	1
15	ZINC000014951634	0.089	0	1	0	0	1
16	ZINC000011616636	0	1	1	1	1	1
17	ZINC000008214697	0	0.003	0	1	0	0
18	ZINC000031165470	1	0	0	0.004	0	0.999
19	ZINC000017654900	1	0	0.816	0	0	0.152
20	ZINC000014951658	1	0	1	0	0	1
21	ZINC000008434966	1	1	0	0	0.993	0.001

Abbreviations: NTP: U.S. National Toxicology Program; DTP: developmental toxicity potential. NTP <0.3 (noncarcinogen); >0.8 (carcinogen). Ames <0.3 (nonmutagen); >0.8 (mutagen). DTP <0.3 (nontoxic); >0.8 (toxic).

### Ligand-binding site analysis and ligand pharmacophore

The structural computation study exhibited the intermolecular interactions between these ligands and CDC20 (Figure 5B and 5C). We use the CDOCKER module of Discovery Studio to assess the ligand binding mechanisms of ZINC000004098930 and apcin with CDC20 (Figure 5D and 5F). And the CDOCKER potential energy of ZINC000004098930 (−29.9471 kcal/mol) was lower than apcin (−25.8556 kcal/mol), which means ZINC000004098930 could bind more firmly than apcin (Table 4). The results also shown the charge, the hydrogen bonds and the π-related interactions between these ligands and CDC20 (Figure 5E, 5G and Supplementary Table 4). It is obvious that ZINC000004098930 had 2 hydrogen bonds and 4 π-related interactions with CDC20, while apcin had only 3 π-related interactions with CDC20.

**Table 4:** CDOCKER potential energy of compounds with CDC20.

**Compounds**	**-CDOCKER Potential Energy (kcal/mol)**
ZINC000004098930	25.8556
ZINC000008434966	29.9471

Then we calculate the pharmacophore of ZINC000004098930 and apcin. The results illustrated that there are 18 features in ZINC000004098930 including 10 hydrogen bond (HB) acceptors, 1 HB-donors, 3 hydrophobics and 4 ring aromatics. While there are 60 features in apcin, including 30 HB acceptors, 23 HB-donors, 3 hydrophobics and 4 ring aromatics (Supplementary Figure 7C and 7D).

### Molecular dynamic simulation

Stability was very significant in drug development, so we use molecular dynamic simulation module to analyze the stability of ZINC000004098930-CDC20 and apcin-CDC20 complexes. We got the potential energy and RMSD curves of these complexes by molecular docking experiment (Figure 5H and 5I). After 18 ps, the ZINC000004098930-CDC20 and apcin-CDC20 complexes’ trajectories reached equilibrium, and gradually being stabilized with time going by. In conclusion, these two complexes could be stable in natural circumstances.

## DISCUSSION

Triple-negative breast cancer (TNBC) as a poor prognosis disease, attracted more and more people’s concern and attention. Owing to lack HR, ER and HER2, TNBC still do not have available target therapy options [20]. In clinical practice, chemotherapy still the main treatment to TNBC patients [21]. Recently, targeted therapy has been a hot topic. And new targeted site and new targeted drugs had been proved very beneficial for tumor patients [22]. Higher response rates were seen when targeted inhibitors are combined with chemotherapy [23]. Therefore, it is very beneficial for us to find new targeted site and new potential ideal targeted drugs.

In this study, we combined bioinformatics with molecular biology to provide new ideas for TNBC treatments. Firstly, we analyzed GSE62931 and GSE76275 database, which contains 245 TNBC tissues and 120 non-TNBC tissues. Trough the Heat maps and Volcano plots, we got 1,212 and 353 differentially expressed genes (DEGs) form these 2 databases. And the Venn plot showed that there are 299 DEGs in both GSE62931 and GSE76275 database, including 88 up-regulated and 141 down-regulated genes. These DEGs could be regarded as potential biomarkers and targeted site for TNBC.

Then, we used metascape website to make the functional enrichment analysis, to study molecular pathways in TNBC. The figures and tables indicated that DEGs were mostly enriched in ‘developmental growth’, ‘regulation of hormone level’ and ‘epithelial cell differentiation’. Also, we respectively analyzed the up-regulated and down-regulated DEGs by functional enrichment in DAVID, and results illustrated that the enrichment of up-regulated DEGs was mainly in BP, MF, CC terms, such as ‘mitotic nuclear division’, ‘identical protein binding’ and ‘cytoplasm’. As for down-regulated DEGs, they were also enriched in BP, MF and CC terms, including ‘negative regulation of cell proliferation’, ‘heme binding’ and ‘extracellular exosome’. In addition, in KEGG pathways, the enrichments of up-regulated and down-regulated DEGs were respectively mostly in ‘cell cycle’ and ‘PPAR signaling pathway’. For example, M. Rath et al. demonstrated that mitotic nuclear division was associated with tumorigenesis, and mitotic kinesins were being validated as drug targets [24, 25]. And extracellular exosome is a vesicle released into extracellular region. Some studies had shown that tumor cells can produce more exosomes than normal cell [26]. Therefore, in short, these pathways were all contribute to the progression of TNBC.

PPI network analysis was very important in bioinformatics research. By the STRING and Cytoscape software, we got the top 20 DEGs as the hub genes based on the degrees. We also got the most significant 3 modules, which could be regarded as the most important gene clusters of TNBC. Among these, module 1 included CDC20 and other 17 genes. We also got the functional pathway enrichment analysis of these 3 modules. DEGs in module 1 were mostly enriched in the BP, CC and KEGG pathway, including ‘spindle’, ‘sister chromatid cohesion’, ‘cell division’, ‘cell cycle’ and ‘mitotic nuclear division’. CDC20 plays a great role in these 5 GO terms and pathway. Meanwhile, most of the DEGs in these 3 modules were enriched in the MF and BP of GO terms, including ‘transcription regulatory region DNA binding’ and ‘structural molecule activity’ and ‘mitotic nuclear division’. On the one hand, it is obvious that abnormal transcription and mitosis could lead to tumorigenesis [27, 28]. On the other hand, there were some studies demonstrated that inhibition of the cellular machinery required for the assembly and maintenance can inhibit the tumor growth [29, 30]. Therefore, these terms and pathways maybe new therapeutic targets for TNBC.

In addition, in Kaplan website, we found 14 hub genes were relevant to OS of TNBC patients, and 19 hub genes were relevant to RFS of TNBC patients. Higher expression of most of them contributed to shorter lifetime, including CDC20, ANLN, ASE1, ASPM, CEP55 and so on. Among these hub genes, cell-division cycle protein 20 homologue (CDC20), as the second important gene based on the degrees in Cytoscape software and the significant gene in module 1, took part in cell division [31]. CDC20, which was key to chromosome segregation and mitosis exit, plays an important role in cell cycle progressing [32]. It can activate a ligase, the anaphase-promoting complex/cyclosome (APC/C), which starts the anaphase and mitotic exit [33]. Cheng et al. shown that overexpression of CDC20 promotes the metastasizing of breast cancer [34]. Meanwhile, some study confirmed that overexpression of CDC20 lead to short-term breast cancer survival again [35]. It was also proved that CDC20 was a great target for anti-tumor drug development [18]. Therefore, CDC20 was a potential treatment target, and we chose CDC20 as a targeted spot for further study.

It is obvious that finding effective CDC20 inhibitor was very important for breast cancer targeted therapy. There were many CDC20 targeted drugs, including tosyl-L-arginine methyl ester (TAME) and apcin, but they still have many issues to be addressed [18]. TAME was proved that can inhibit the binding of free CDC20 and APC and promote the CDC20 removal from the APC [36, 37]. And apcin can bind to CDC20 and simultaneously disrupt the APC/C-Cdc20-substrate ternary complex by competitively inhibition to blockade the mitotic exit [38]. It was also proved that apcin can inhibit the growth and invasion of osteosarcoma cell by targeting CDC20 [39]. However, whether TAME and apcin were useful in clinical needs further investments. Among these CDC20 inhibitor, we chose apcin as the reference drug to screen new potential ideal compounds for TNBC patients.

We got 7,416 natural ligands by LibDock, and based on the LibDock score, we chose top 20 ligands to do the further study. Safety is one of the most important things in drug development. Therefore, after analyzing their biochemico-pharmacological properties by ADME and Toxicity Prediction module, we chose ZINC000004098930, which was non-hepatotoxicity, more solubility level and less carcinogenicity than apcin, as the safe lead compounds among the top 20 ligands.

Then we analyzed the pharmacophore and the ligand binding mechanisms of ZINC000004098930 and apcin with CDC20. The results demonstrated that the CDOCKER potential energy of ZINC000004098930 was lower than apcin, which means ZINC000004098930 could bind more firmly than apcin. Meanwhile, ZINC000004098930 had more hydrogen bonds and the π-related interactions with CDC20 than apcin. In general, ZINC000004098930 had a higher binding force with CDC20 than apcin.

In the end, we run RMSD and calculated the potential energy of ZINC000004098930-CDC20 and apcin-CDC20 complexes to study the stability of them by molecular dynamics simulation. As the results suggested, the trajectories of both ZINC000004098930-CDC20 and apcin-CDC20 complexes reached their equilibrium after 18 ps. They become gradually stabilized, which indicated these two complexes could exist stability in natural. In conclusion, ZINC000004098930 could be regarded as ideal lead compounds for drug development for TNBC patients and may give new thoughts to TNBC targeted therapy.

Recently, targeted therapy is a hot topic for tumor treatment, but we still do not have perfect drugs for TNBC treatment. In this study, we combined bioinformatics with molecular biology to screen a new ideal ligand, which targeted inhibit CDC20. Although there is a long way from clinical application, it provided a new way to treat TNBC. ZINC000004098930 as a natural ligand has unique advantages. To sum up, we did the first step of drug development for TNBC patients. And what’s more, we provided 18 else hub genes and many targeted pathways, which may be useful in future study.

## CONCLUSIONS

We found 229 DEGs between TNBC tissues and non-TNBC tissues, including 88 up-regulated and 141 down-regulated DEGs. 20 hub genes were screened and most of them were relevant to the survival time of breast cancer patients. Therefore, we chose CDC20, which plays a great role in TNBC, as the potential target. We screened 7,416 natural ligands that can bind firmly with CDC20 from ZINC database. And among these ligands, ZINC000004098930 was regarded as the potential ideal ligand, owing to its non-hepatotoxicity, more solubility level and less carcinogenicity than the reference drug, apcin. Meanwhile, ZINC000004098930-CDC20 was proved could exist stably in natural environment. In short, ZINC000004098930 may be the ideal targeted ligand after modification, which may give great contribution to TNBC targeted treatment.

## Supplementary Materials

Supplementary Figures

Supplementary Tables
