# Supplementary material for: Selected ideal natural ligand against TNBC by inhibiting CDC20, using bioinformatics and molecular biology
Source: Aging (Albany NY). 2021 Oct 22;13(20):23702–25. doi: 10.18632/aging.203642 (PMC8580355; doi:10.18632/aging.203642)
Supplement: Supplementary Figures [file aging-13-203642-s001.pdf]

SUPPLEMENTARY FIGURES

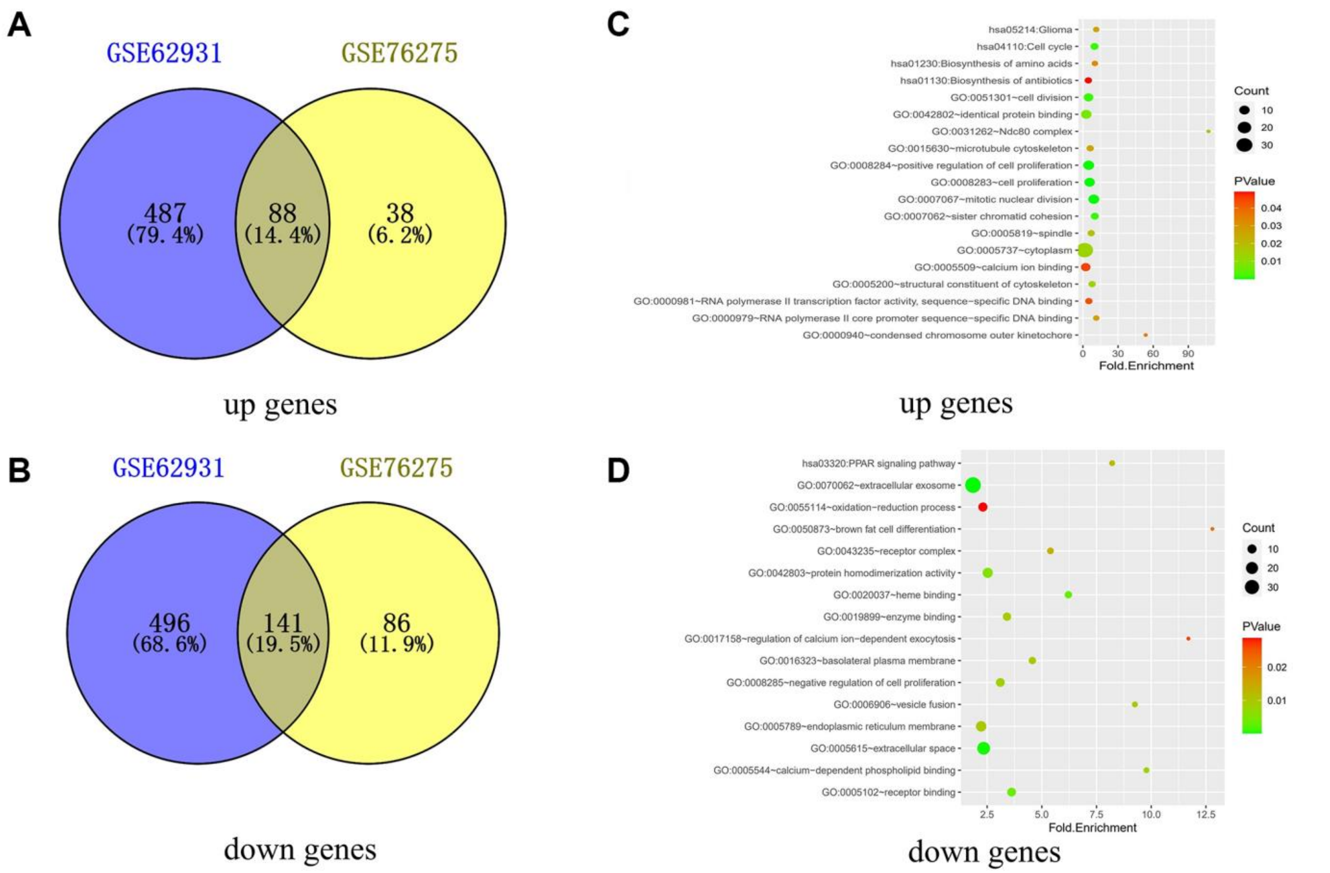

Supplementary Figure 1. (**A**) Venn plot of up-regulated DEGs in GSE62931 and GSE76275. (**B**) Venn plot of down-regulated DEGs in GSE62931 and GSE76275. (**C**) Functional annotation and enrichment of up-regulated DEGs. (**D**) Functional annotation and enrichment of down-regulated DEGs.

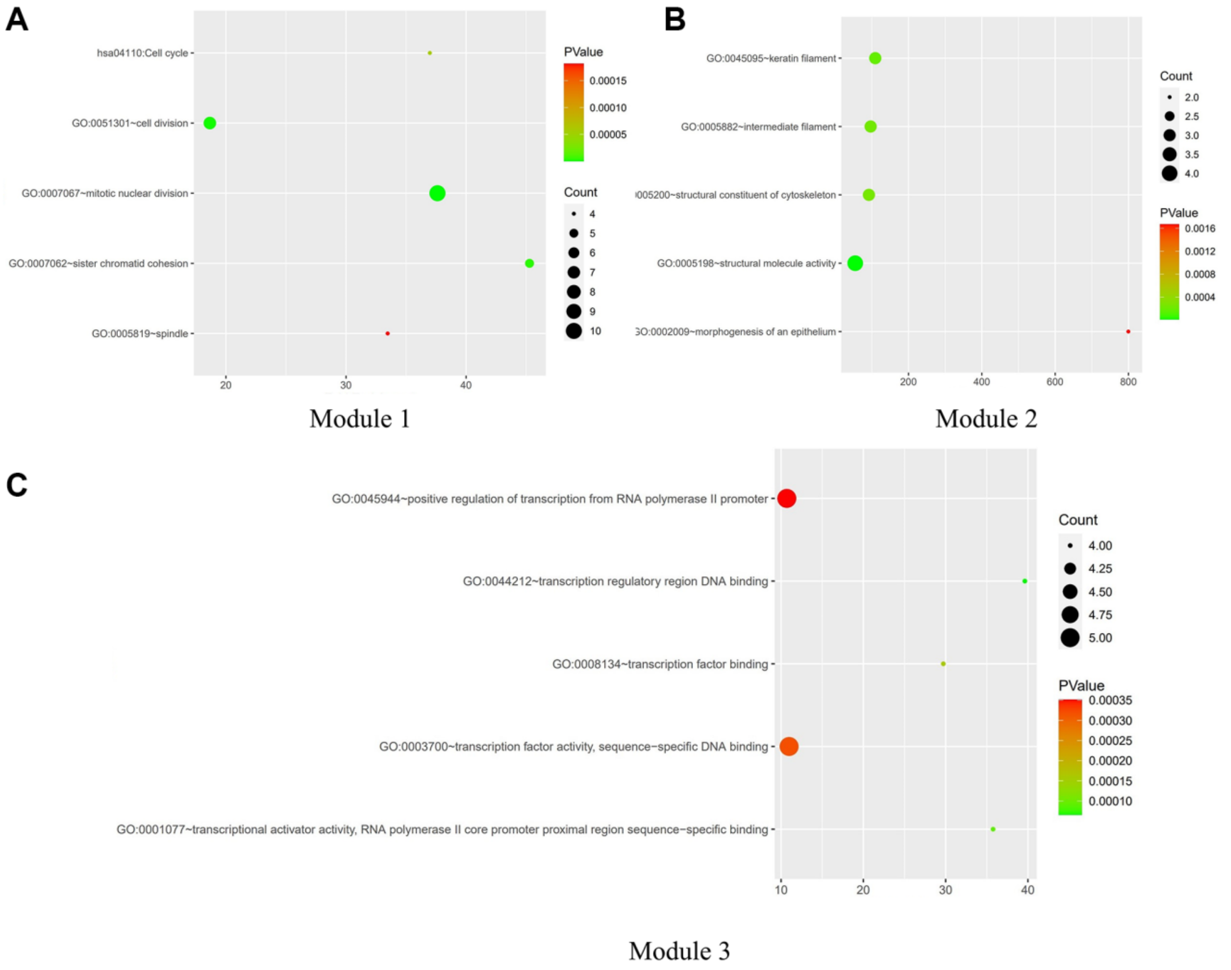

Supplementary Figure 2. Functional annotation and enrichment of (**A**) Module 1 (**B**) Module 2 (**C**) Module 3.

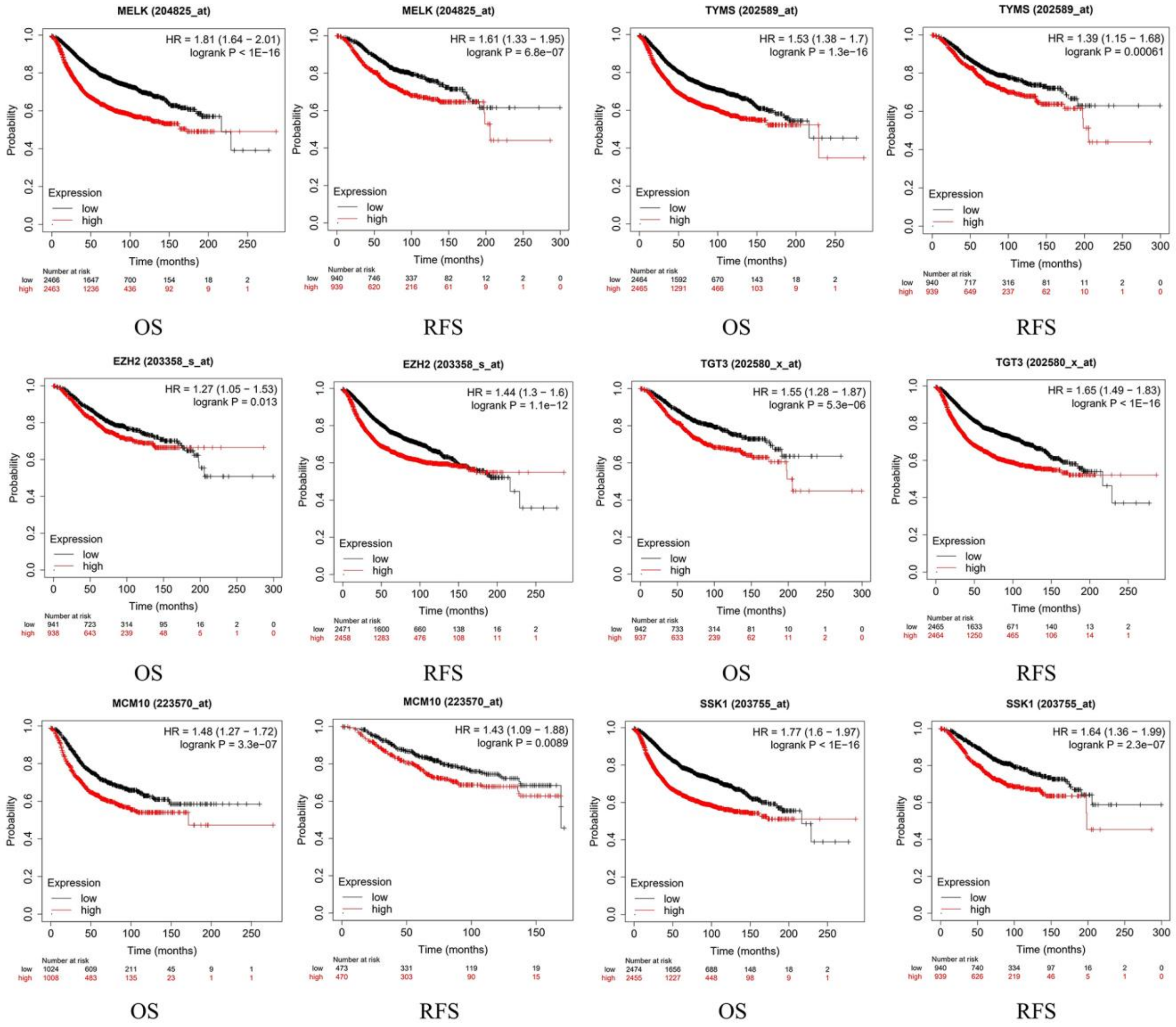

Supplementary Figure 3. The OS and RFS of 19 hub genes except CDC20.

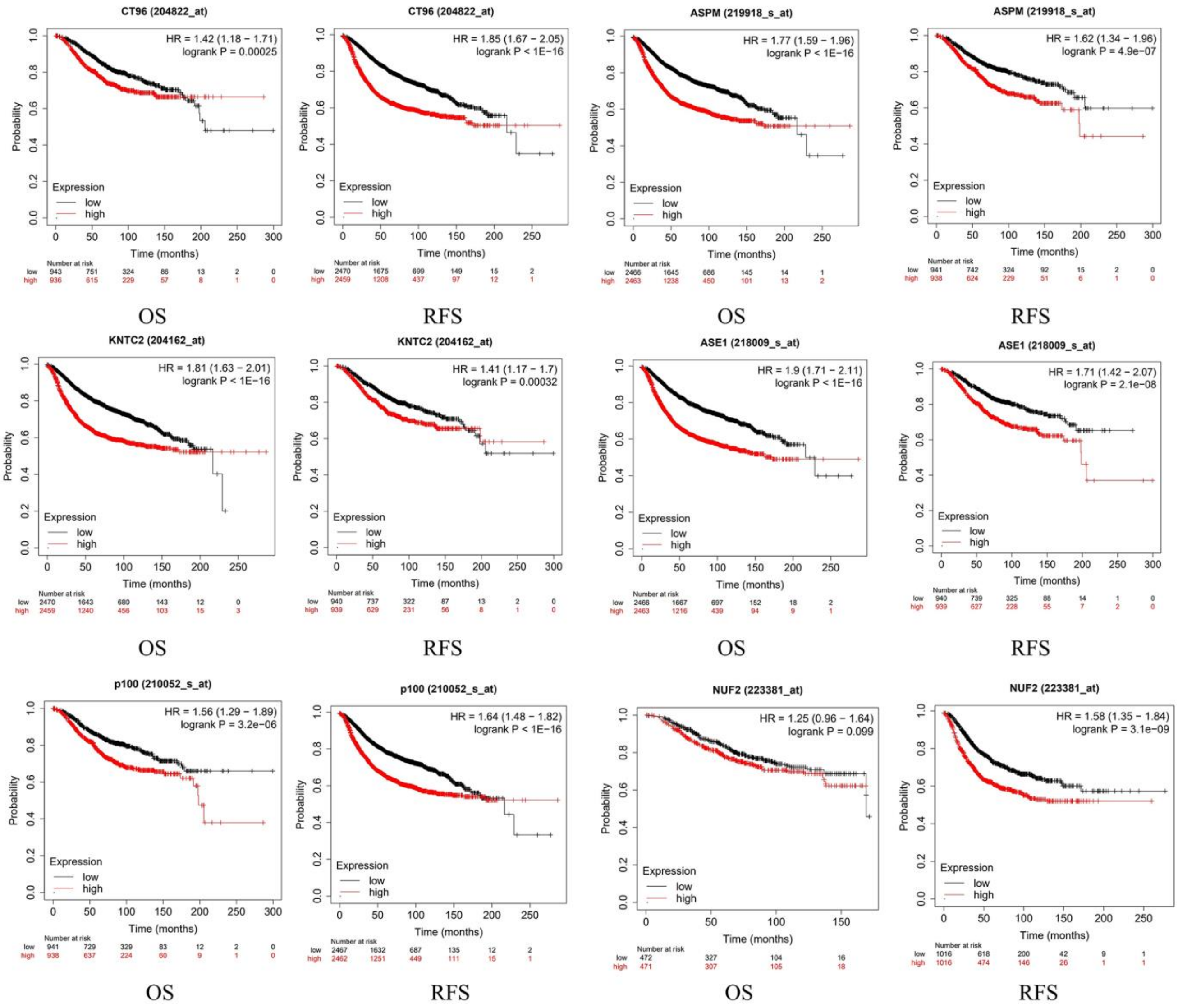

Supplementary Figure 4. The OS and RFS of 19 hub genes except CDC20.

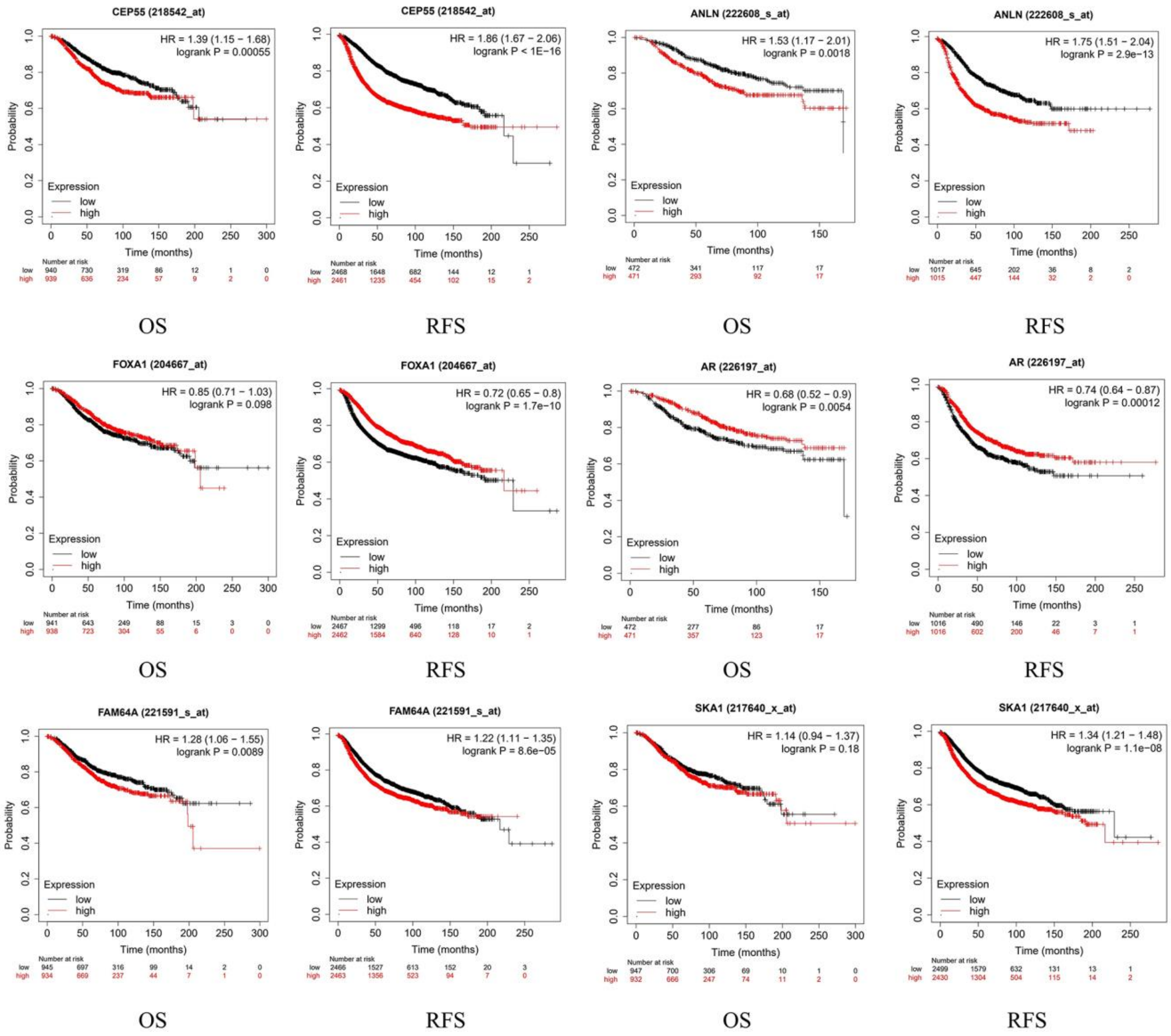

Supplementary Figure 5. The OS and RFS of 19 hub genes except CDC20.

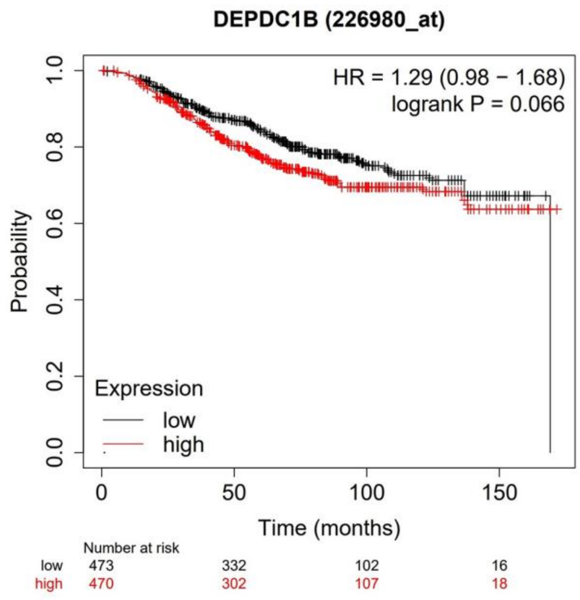

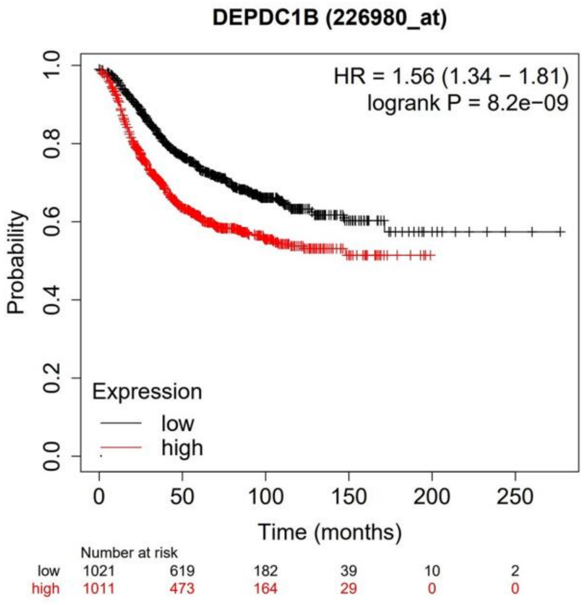

Supplementary Figure 6. The OS and RFS of 19 hub genes except CDC20.

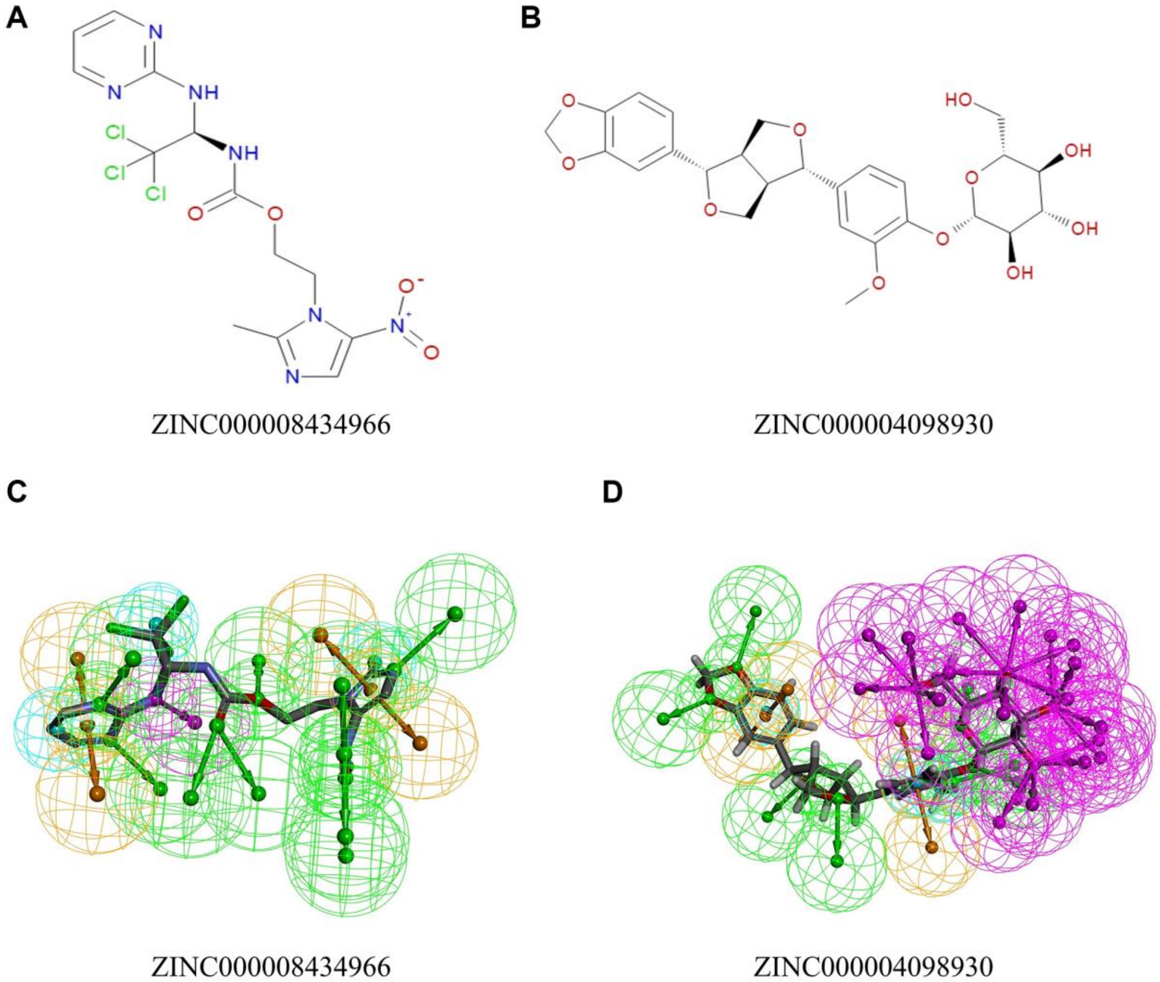

Supplementary Figure 7. (**A**) Chemical structures of ZINC000008434966. (**B**) Chemical structures of ZINC000004098930. (**C**) Results of the pharmacophore of the ZINC000008434966. (**D**) Results of the pharmacophore of the ZINC000004098930.
