# Supplementary material for: Selected ideal natural ligand against TNBC by inhibiting CDC20, using bioinformatics and molecular biology
Source: Aging (Albany NY). 2021 Oct 22;13(20):23702–25. doi: 10.18632/aging.203642 (PMC8580355; doi:10.18632/aging.203642)
Supplement: Supplementary Tables [file aging-13-203642-s002.pdf]

SUPPLEMENTARY TABLES

Supplementary Table 1. Functional and pathway enrichment analysis of up-regulated and down-regulated genes.

Expression	Category	Term	Count	%	*P* Value
up-regulated	GOTERM_BP_DIRECT	GO:0007067~mitotic nuclear division	11	12.64368	2.43E-07
	GOTERM_BP_DIRECT	GO:0008283~cell proliferation	10	11.49425	5.40E-05
	GOTERM_BP_DIRECT	GO:0008284~positive regulation of cell proliferation	11	12.64368	6.39E-05
	GOTERM_BP_DIRECT	GO:0051301~cell division	8	9.195402	0.00129
	GOTERM_BP_DIRECT	GO:0007062~sister chromatid cohesion	5	5.747126	0.00141
	GOTERM_CC_DIRECT	GO:0005737~cytoplasm	35	40.22989	0.01308
	GOTERM_CC_DIRECT	GO:0031262~Ndc80 complex	2	2.298851	0.01831
	GOTERM_CC_DIRECT	GO:0005819~spindle	4	4.597701	0.01843
	GOTERM_CC_DIRECT	GO:0015630~microtubule cytoskeleton	4	4.597701	0.02546
	GOTERM_CC_DIRECT	GO:0000940~condensed chromosome outer kinetochore	2	2.298851	0.03629
	GOTERM_MF_DIRECT	GO:0042802~identical protein binding	10	11.49425	0.00691
	GOTERM_MF_DIRECT	GO:0005200~structural constituent of cytoskeleton	4	4.597701	0.01389
	GOTERM_MF_DIRECT	GO:0000979~RNA polymerase II core promoter	3	3.448276	0.02788
	GOTERM_MF_DIRECT	GO:0000981~RNA polymerase II transcription factor activity	4	4.597701	0.04333
	GOTERM_MF_DIRECT	GO:0005509~calcium ion binding	8	9.195402	0.04485
	KEGG_PATHWAY	hsa04110:Cell cycle	5	5.747126	0.00128
	KEGG_PATHWAY	hsa05214:Glioma	3	3.448276	0.02651
	KEGG_PATHWAY	hsa01230:Biosynthesis of amino acids	3	3.448276	0.03204
	KEGG_PATHWAY	hsa01130:Biosynthesis of antibiotics	4	4.597701	0.04903
down-regulated	GOTERM_BP_DIRECT	GO:0008285~negative regulation of cell proliferation	9	6.428571	0.00833
	GOTERM_BP_DIRECT	GO:0006906~vesicle fusion	4	2.857143	0.00905
	GOTERM_BP_DIRECT	GO:0050873~brown fat cell differentiation	3	2.142857	0.02253
	GOTERM_BP_DIRECT	GO:0017158~regulation of calcium ion-dependent exocytosis	3	2.142857	0.02665
	GOTERM_BP_DIRECT	GO:0055114~oxidation-reduction process	10	7.142857	0.02866
	GOTERM_CC_DIRECT	GO:0070062~extracellular exosome	38	27.14286	0.00015
	GOTERM_CC_DIRECT	GO:0005615~extracellular space	23	16.42857	0.00026
	GOTERM_CC_DIRECT	GO:0005789~endoplasmic reticulum membrane	14	10	0.00977
	GOTERM_CC_DIRECT	GO:0016323~basolateral plasma membrane	6	4.285714	0.01004
	GOTERM_CC_DIRECT	GO:0043235~receptor complex	5	3.571429	0.01371
	GOTERM_MF_DIRECT	GO:0020037~heme binding	6	4.285714	0.00275
	GOTERM_MF_DIRECT	GO:0005102~receptor binding	9	6.428571	0.00337
	GOTERM_MF_DIRECT	GO:0042803~protein homodimerization activity	13	9.285714	0.00505
	GOTERM_MF_DIRECT	GO:0005544~calcium-dependent phospholipid binding	4	2.857143	0.00777
	GOTERM_MF_DIRECT	GO:0019899~enzyme binding	8	5.714286	0.00891
	KEGG_PATHWAY	hsa03320:PPAR signaling pathway	4	2.857143	0.01182

Supplementary Table 2. Functional and pathway enrichment analysis of the module genes.

Module	Term	Count	*P* Value	Genes	FDR
Module1	GO:0007067~mitotic nuclear division	10	6.34E-13	CDC20, ASPM, ANLN, TPX2, NUF2, BUB1B, FAM64A, NDC80, SKA1, CEP55	9.89E-11
	GO:0051301~cell division	7	8.01E-07	CDC20, TPX2, NUF2, BUB1B, FAM64A, NDC80, SKA1	6.25E-05
	GO:0007062~sister chromatid cohesion	5	2.99E-06	CDC20, NUF2, BUB1B, NDC80, SKA1	0.000155385
	hsa04110:Cell cycle	4	5.57E-05	CDC20, ORC1, BUB1B, TTK	0.000501195
	GO:0005819~spindle	4	0.000181	CDC20, TPX2, PRC1, TTK	0.007255614
Module2	GO:0005198~structural molecule activity	4	1.22E-05	KRT81, KRT16, KRT23, KRT6A	2.45E-05
	GO:0045095~keratin filament	3	0.000178	KRT81, KRT6B, KRT6A	0.000793813
	GO:0005882~intermediate filament	3	0.000227	KRT16, KRT23, KRT6A	0.000793813
	GO:0005200~structural constituent of cytoskeleton	3	0.00025	KRT16, KRT6B, KRT6A	0.000250315
	GO:0002009~morphogenesis of an epithelium	2	0.001667	KRT16, KRT6A	0.016072382
Module3	GO:0044212~transcription regulatory region DNA binding	4	6.68E-05	FOXA1, AR, FOXC1, GATA3	0.001494749
	GO:0001077~transcriptional activator activity	4	9.06E-05	FOXA1, AR, PGR, GATA3	0.001494749
	GO:0008134~transcription factor binding	4	0.000157	FOXA1, AR, FOXC1, GATA3	0.001725422
	GO:0003700~transcription factor activity, sequence-specific DNA binding	5	0.000318	FOXA1, AR, FOXC1, PGR, GATA3	0.002623593
	GO:0045944~positive regulation of transcription from RNA polymerase II promoter	5	0.000351	FOXA1, AR, FOXC1, PGR, GATA3	0.078369289

Supplementary Table 3. CDC20 targeted drugs downloaded from ZICN15 database.

Number	Compounds	Libdock score
1	ZINC000001577210	117.193
2	ZINC000014455080	118.871
3	ZINC000085826837	121.348
4	ZINC000013130935	117.227
5	ZINC000004098930	119.045
6	ZINC000028968101	135.361
7	ZINC000028968107	120.747
8	ZINC000044281738	130.698
9	ZINC000038143594	140.287
10	ZINC000044086691	129.699
11	ZINC000002526388	118.787
12	ZINC000049784088	132.543
13	ZINC000004098458	123.630
14	ZINC000004098459	118.383
15	ZINC000014951634	128.112
16	ZINC000011616636	124.048
17	ZINC000008214697	130.294
18	ZINC000031165470	119.017
19	ZINC000017654900	129.874
20	ZINC000014951658	134.454

Supplementary Table 4. Hydrogen bond interaction parameters and π-related interaction parameters for each compound with the receptor CDC20.

Compound	Interaction Parameters	Donor Atom	Receptor Atom	Distances (Å)
ZINC000004098930	π-interaction parameters	ILE216	ZINC000004098930	4.90
		ZINC000004098930	LEU176	5.06
		ZINC000004098930	VAL200	4.11
ZINC000008434966	Hydrogen bond interaction	ZINC000008434966:H32	ASP177:O	2.55
		ZINC000008434966:H33	ASP177:O	2.48
	π-interaction parameters	ZINC000008434966:C1	ILE216	4.06
		TYR207	ZINC000008434966:C1	4.48
		ZINC000008434966	VAL200	1.60
		ZINC000008434966	ILE216	4.69
